# Supplementary figures and images for: Piezo1-mediated spontaneous calcium transients in satellite glia impact dorsal root ganglia development
Source: PLoS Biol. 2023 Sep 25;21(9):e3002319. doi: 10.1371/journal.pbio.3002319 (PMC10564127; doi:10.1371/journal.pbio.3002319)

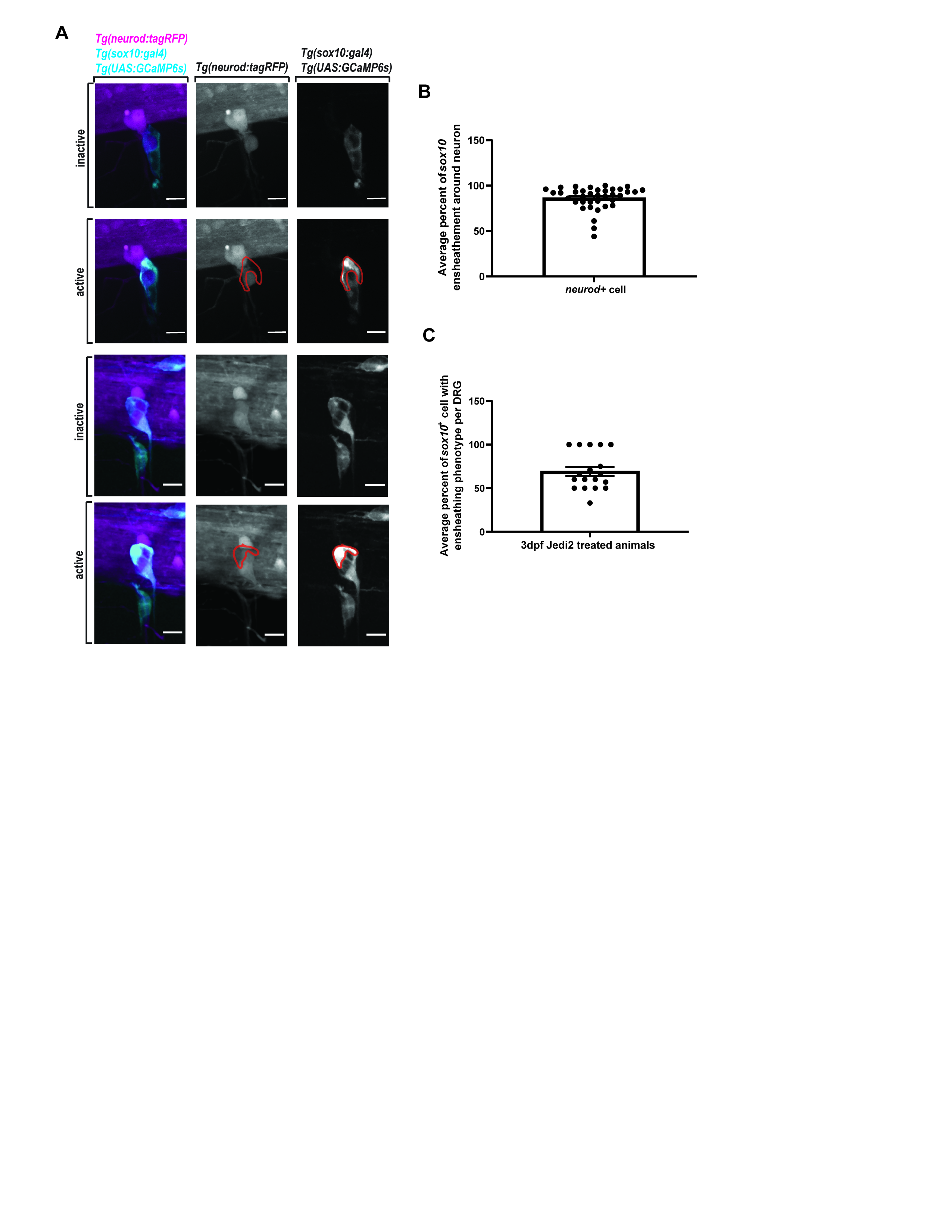

Supplement: S1 Fig — (A) Confocal z-projections of DRG in 3 dpf animals expressing Tg(sox10:gal4+myl7); Tg(uas:GCaMP6s); Tg(neurod:tagRFP). Magenta displays neurod+ neurons. Cyan displays sox10+ satellite glia. Red tracing indicates the morphology of a satellite glia during a Ca2+ transient event. (B) Quantification of the average percent of sox10+ satellite glia ensheathment around a neurod+ neuron at 3 dpf (n = 5 animals, 14 DRG, 37 cells). (C) Quantification of the average number of sox10+ cells with an ensheathing morphology following 40 μM Jedi2 treatment at 3 dpf (7 animals, 18 DRG, 74 cells). Scale bar is 10 μM (A). The data underlying this figure can be found in S1 Data. (TIF) [file pbio.3002319.s001.tif]

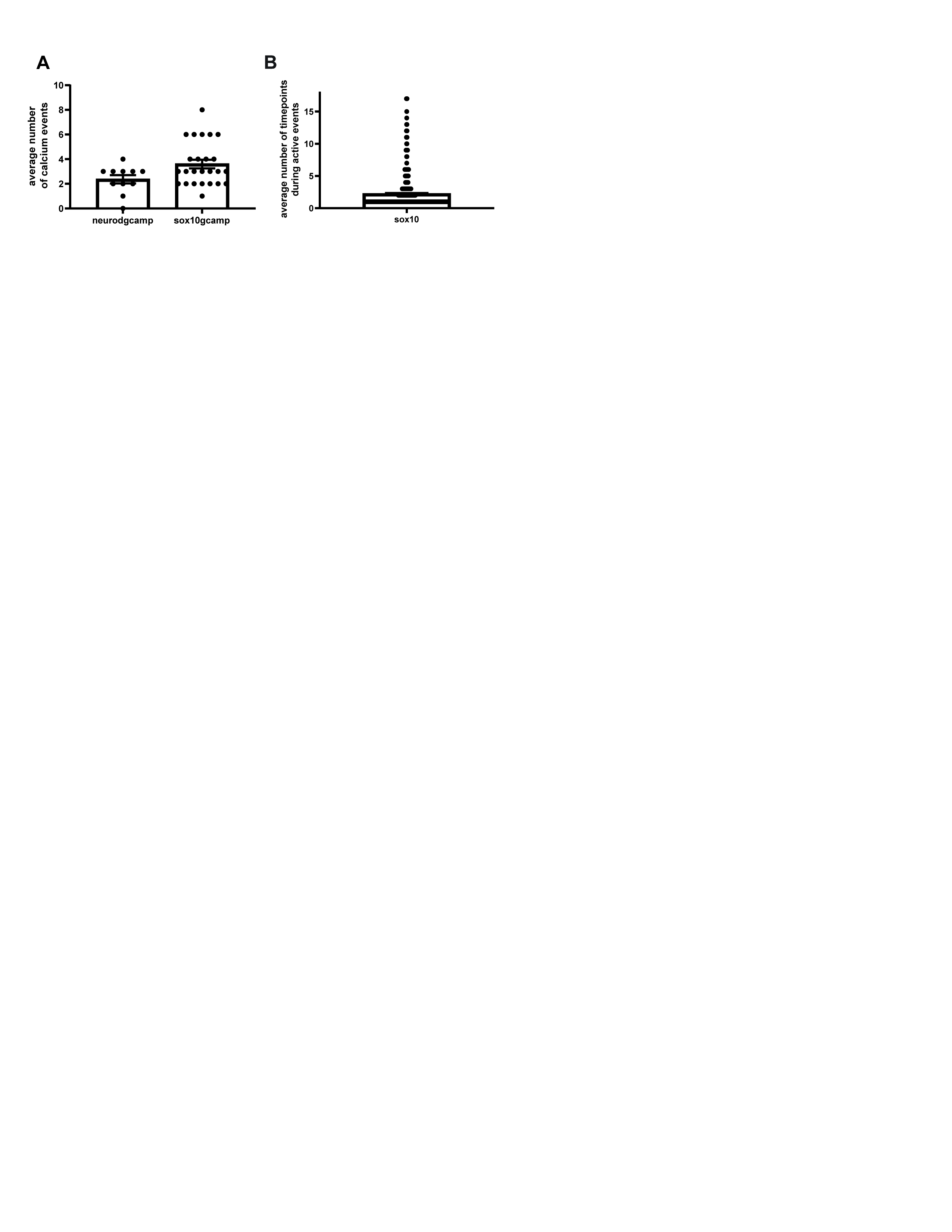

Supplement: S2 Fig — (A) Quantification of the average number of calcium events per neurod+ cell or per sox10+ cell in 3 dpf animals expressing either Tg(neurod:gal4+myl7); Tg(uas:GCaMP6s) or Tg(sox10:gal4+myl7); Tg(uas:GCaMP6s) (neurod: n = 4 animals, 9 DRG, 11 cells, sox10: n = 5 animals, 14 DRG, 25 cells). (B) Quantification of the average number of time points during active events per sox10+ cells in animals expressing Tg(sox10:gal4+myl7); Tg(uas:GCaMP6s) (n = 5 animals, 20 DRG, 97 cells, 412 calcium transient events). The data underlying this figure can be found in S1 Data. (TIF) [file pbio.3002319.s002.tif]

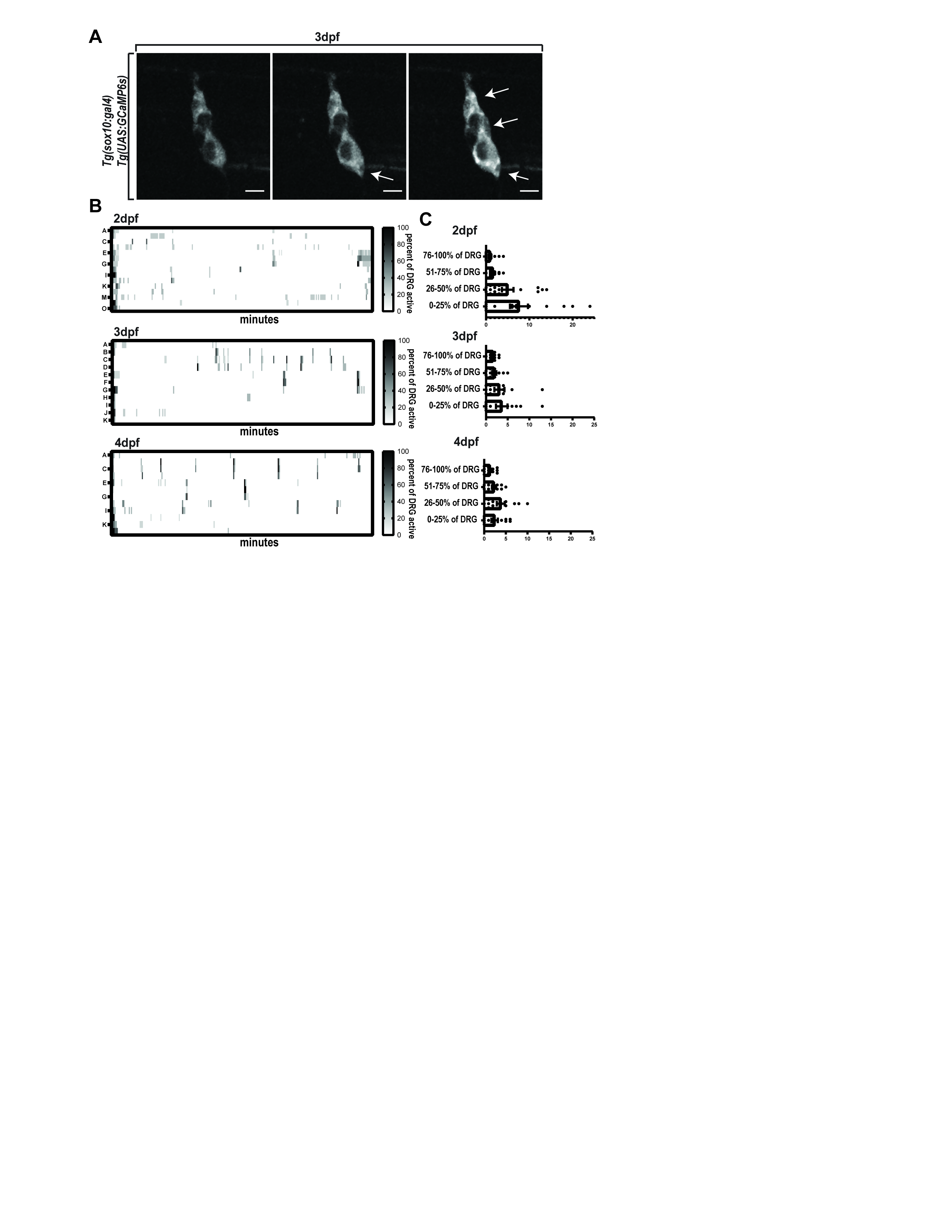

Supplement: S3 Fig — (A) Confocal z-projection of DRG in 3 dpf animals expressing Tg(sox10:gal4+myl7); Tg(uas:GCaMP6s). Arrows note active cells and demonstrate different percentages of active DRG. (B) Heatmaps of the percent of cells in the DRG active during a 1-h period at 2, 3, and 4 dpf. Darker gradient indicates a higher percent of cells active (2 dpf: n = 6 animals, 10 DRG, 46 cells, 3 dpf: n = 4 animals, 6 DRG, 27 cells, 4 dpf: n = 4 animals, 7 DRG, 34 cells). (C) Quantification of the number of active events with 0%–25%, 26%–50%, 51%–75%, or 76%–100% of DRG cells active at the same time point at 2, 3, and 4 dpf (2 dpf: n = 6 animals, 10 DRG, 46 cells, 3 dpf: n = 4 animals, 6 DRG, 27 cells, 4 dpf: n = 4 animals, 7 DRG, 34 cells). Scale bar is 10 μM (A). The data underlying this figure can be found in S1 Data. (TIF) [file pbio.3002319.s003.tif]

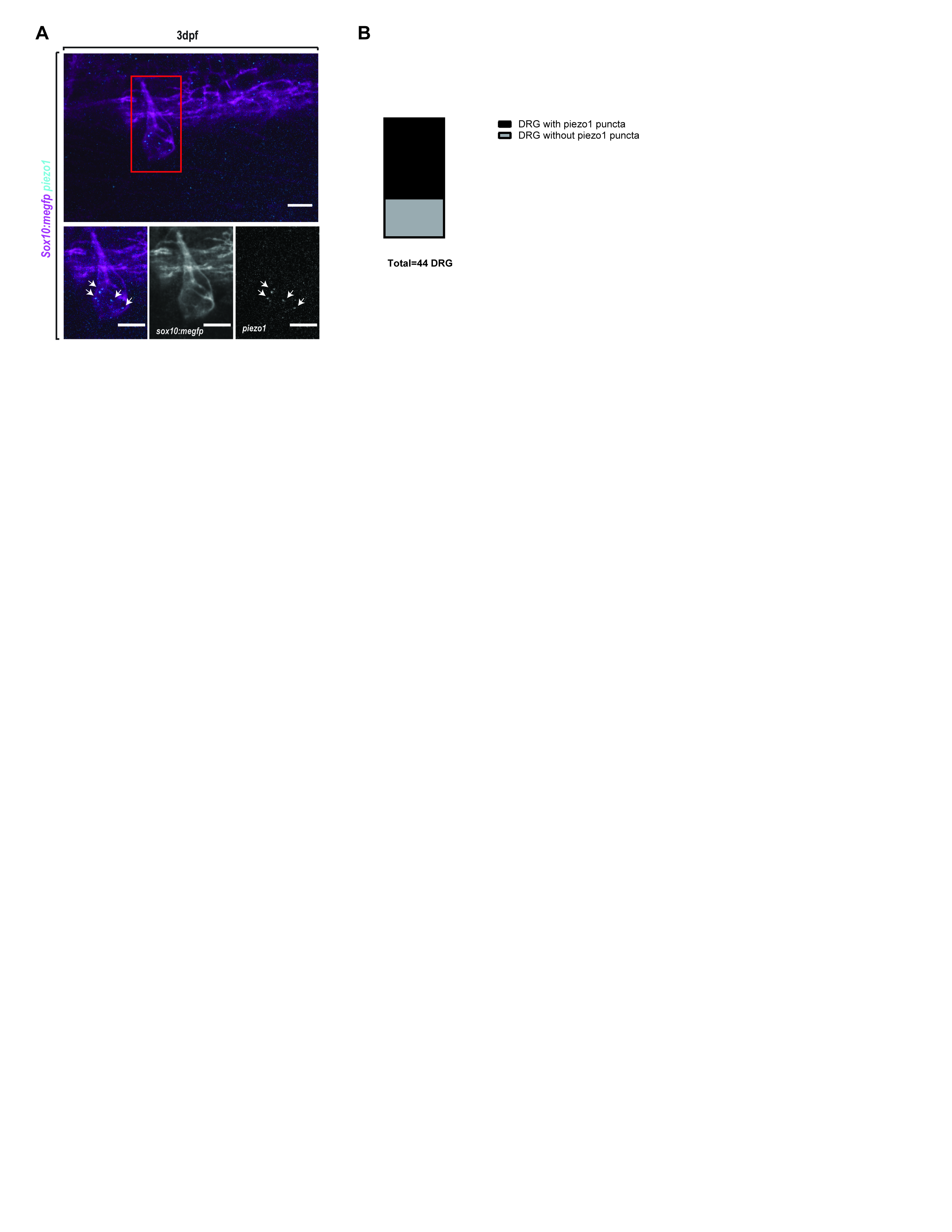

Supplement: S4 Fig — (A) Confocal images of HCR-FISH-piezo1 and Immunohistochemistry-GFP in 3 dpf Tg(sox10:meGFP) animals. GFP is shown in magenta and piezo1 is shown in cyan. Arrows indicate piezo1 puncta. (B) Quantification of percent of DRG at 3 dpf with piezo1 puncta and without piezo1 puncta (n = 13 animals, 44 DRG). Scale bar is 10 μM (A). The data underlying this figure can be found in S1 Data. (TIF) [file pbio.3002319.s004.tif]

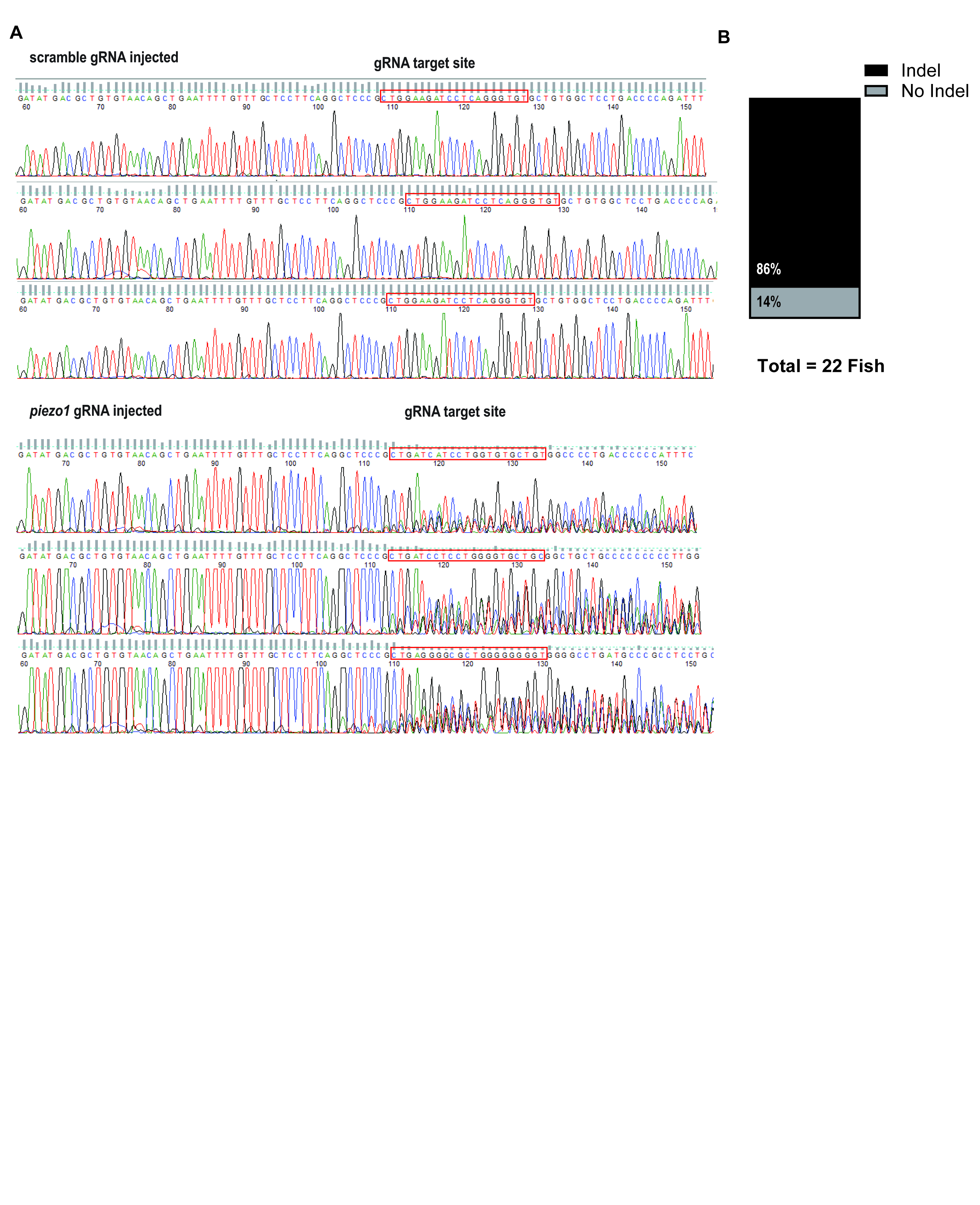

Supplement: S5 Fig — (A) Sanger sequencing of 3 individual animals following injection with a scrambled gRNA along with Cas9 (Top). Sanger sequencing of 3 individual animals following injection with a piezo1 gRNA along with Cas9 (Bottom). Red box indicates target site of piezo1 gRNA. (B) Quantification of the percent of animals with indels according to Sanger sequencing data in animals injected with piezo1 gRNA and Cas9 (n = 22 animals). The data underlying this figure can be found in S1 Data. (TIF) [file pbio.3002319.s005.tif]

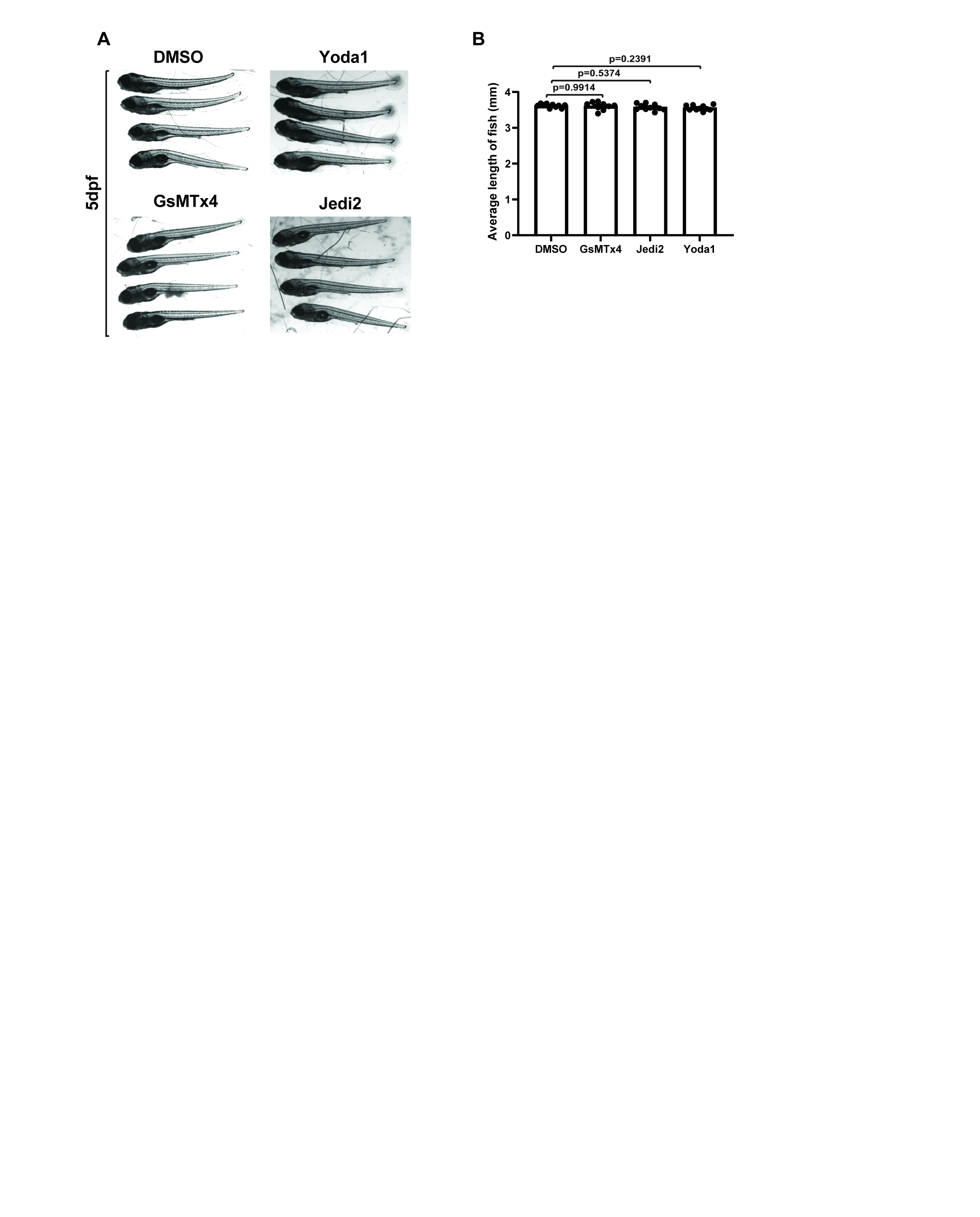

Supplement: S6 Fig — (A) Images of 5 dpf animals following treatments of DMSO, GsMTx4, Yoda1, or Jedi2. (B) Quantifications of the average length of animals (mm) following consecutive days of pharmacological treatment (DMSO: n = 10 animals, GsMTx4: n = 9 animals, Jedi2: n = 10 animals, Yoda1: n = 10 animals). The data underlying this figure can be found in S1 Data. (TIF) [file pbio.3002319.s006.tif]

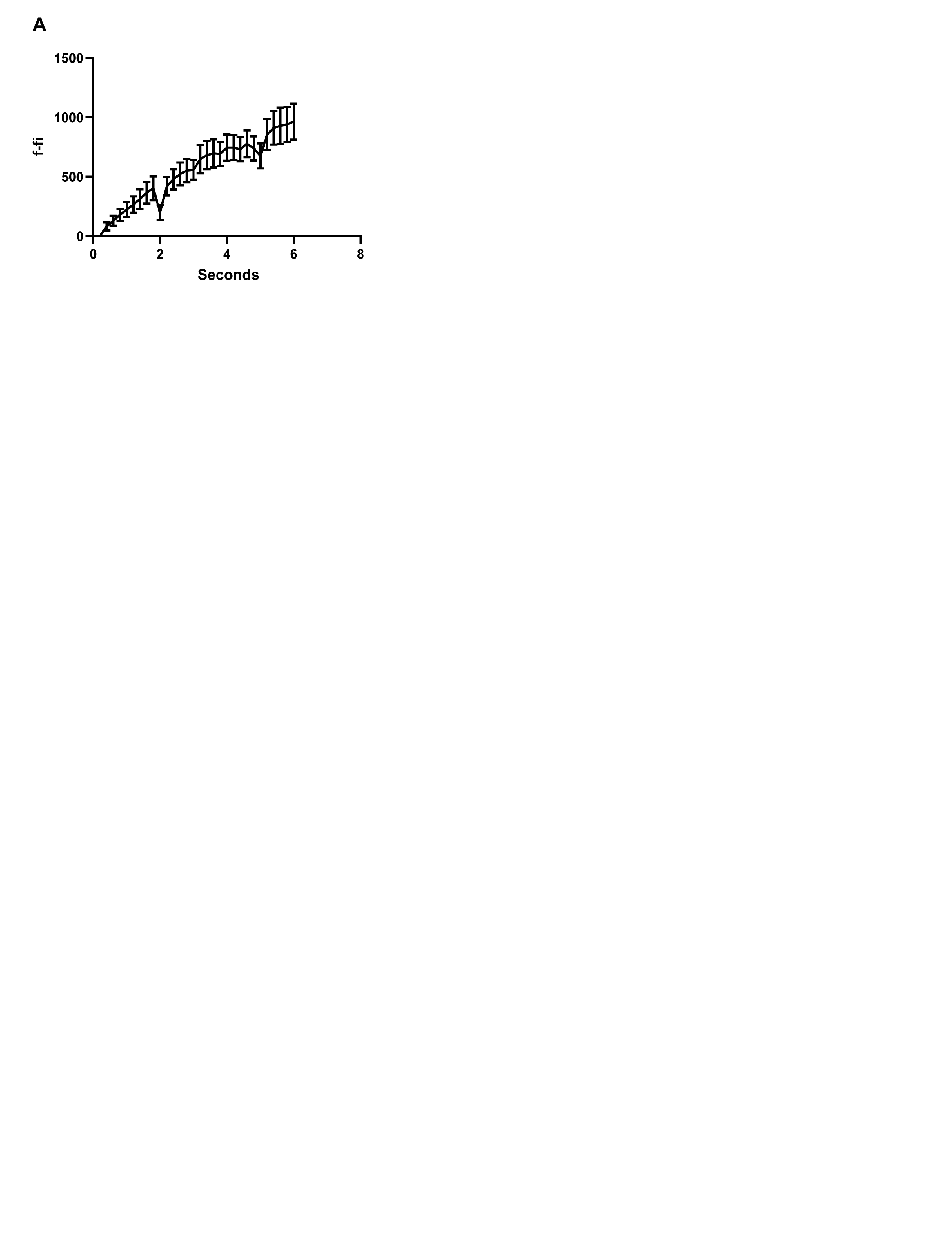

Supplement: S7 Fig — (A) Quantification of the average change in integral density of fluorescence in sox10+ cells over time (seconds) following activation of cochr2 with 488 nm light at 3 dpf. Change in fluorescence was measured by subtracting the initial integral density of fluorescence from each time point (n = 7 animals, 7 DRG, 7 cells). The data underlying this figure can be found in S1 Data. (TIF) [file pbio.3002319.s007.tif]

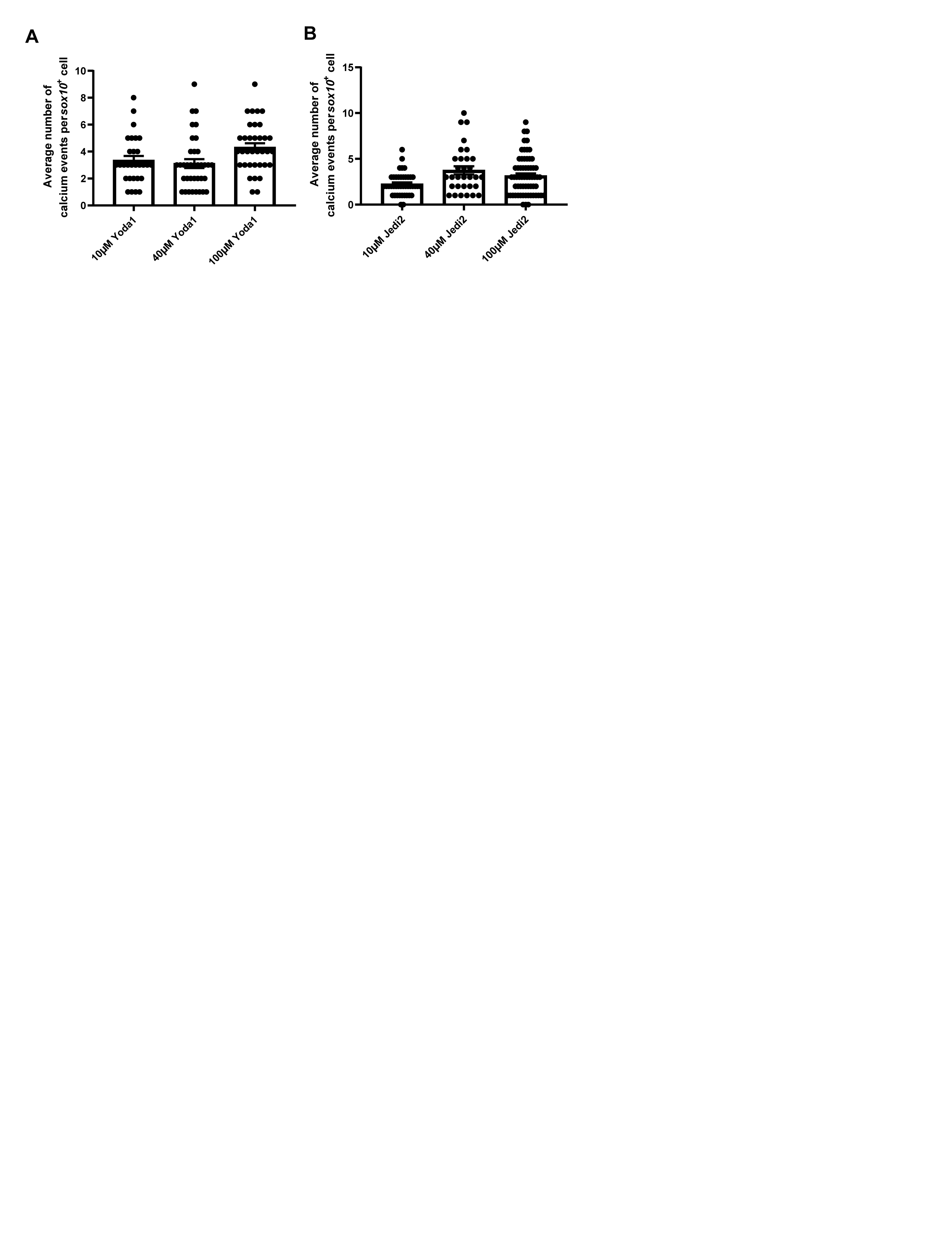

Supplement: S8 Fig — (A) Quantification of the average number of Ca2+ transient events in sox10+ cells of 3 dpf animals expressing Tg(sox10:gal4+myl7); Tg(uas:GCaMP6s); Tg(neurod:tagRFP) treated with 10 μM, 40 μM, or 100 μM Yoda1 in 2% DMSO for 30 min prior to imaging (10 μM: n = 2 animals, 7 DRG, 29 cells, 40 μM: n = 3 animals, 9 DRG, 36 cells, 100 μM: n = 2 animals, 5 DRG, 35 cells). (B) Quantification of the average number of Ca2+ transient events in sox10+ cells of 3 dpf animals expressing Tg(sox10:gal4+myl7); Tg(uas:GCaMP6s); Tg(neurod:tagRFP) treated with 10 μM, 40 μM, or 100 μM Jedi2 in 2% DMSO for 30 min prior to imaging (10 μM: n = 3 animals, 9 DRG, 42 cells, 40 μM: n = 3 animals, 8 DRG, 31 cells, 100 μM: n = 5 animals, 14 DRG, 63 cells). The data underlying this figure can be found in S1 Data. (TIF) [file pbio.3002319.s008.tif]

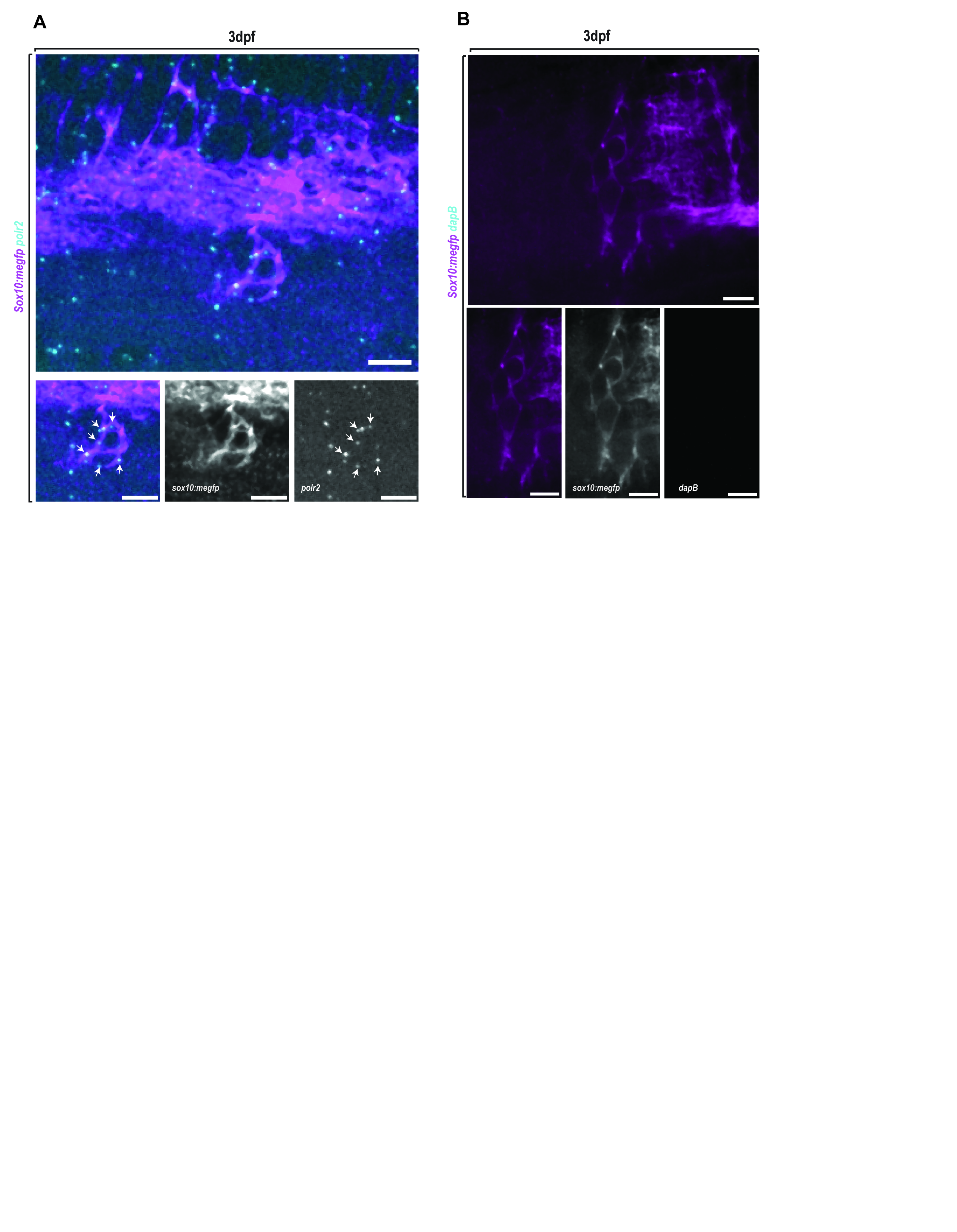

Supplement: S9 Fig — (A) Confocal images of ubiquitous RNAscope probe polr2 and Immunohistochemistry-GFP in 3 dpf Tg(sox10:meGFP) animals. GFP is shown in magenta and polr2 is shown in cyan. Arrows indicate polr2 puncta (scale bar is 10 μM). (B) Confocal images of bacterial RNAscope probe dapB and Immunohistochemistry-GFP in 3 dpf Tg(sox10:meGFP) animals. GFP is shown in magenta and dapB is shown in cyan. Arrows indicate dapB puncta (scale bar is 10 μM). (TIF) [file pbio.3002319.s009.tif]

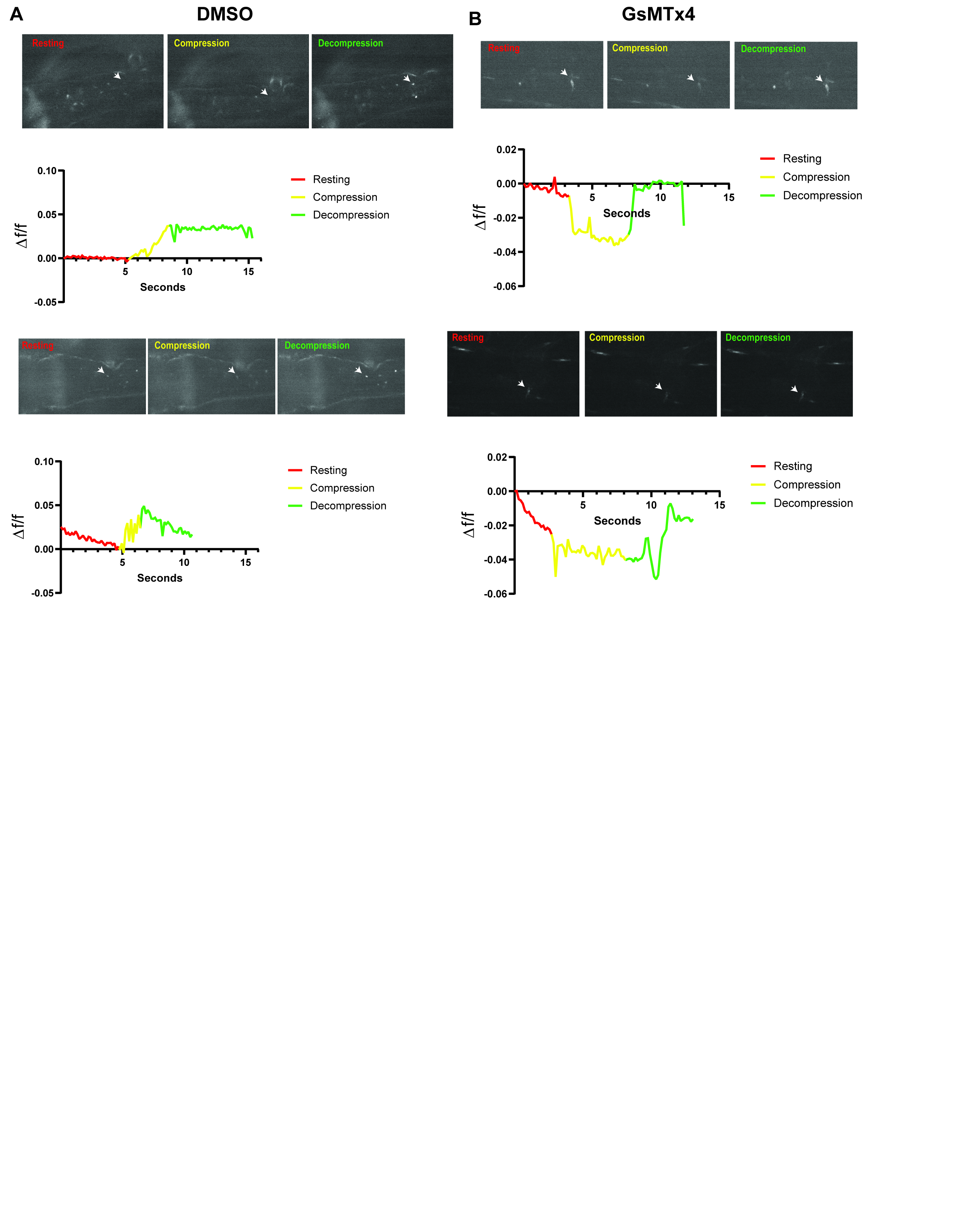

Supplement: S10 Fig — (A) Confocal images of 3 dpf animals treated with 2% DMSO expressing Tg(sox10:gal4); Tg(uas:GCaMP6s) during resting phase, compression phase, and decompression phase. Quantifications of the change of fluorescence during each phase is shown below images. (B) Confocal images of 3 dpf animals treated with 1 μM GsMTx4 expressing Tg(sox10:gal4); Tg(uas:GCaMP6s) during resting phase, compression phase, and decompression phase. Quantifications of the change of fluorescence during each phase is shown below images. Arrows in all images identify DRG being measured during the assay. The data underlying this figure can be found in S1 Data. (TIF) [file pbio.3002319.s010.tif]
